# Association of signalment parameters with activity of pet dogs*

**DOI:** 10.1017/jns.2014.49

**Published:** 2014-09-25

**Authors:** Kathryn E. Michel, Dorothy C. Brown

**Affiliations:** 1 Department of Clinical Studies – Philadelphia, School of Veterinary Medicine, University of Pennsylvania, 3900 Delancey Street, Philadelphia, PA 19104-6010, USA; 2 Department of Clinical Studies – Philadelphia, School of Veterinary Medicine, 3900 Delancey Street, Philadelphia, PA 19104-6010, USA, and Center for Clinical Epidemiology and Biostatistics, School of Medicine, University of Pennsylvania, 423 Guardian Drive, Philadelphia, PA 19104-6021, USA

**Keywords:** Canine nutrition, Dogs, Physical activity, Accelerometry

## Abstract

Activity monitors are increasingly being used to quantify the activity of pet dogs. The aim of the present study was to investigate associations between signalment and activity of free-living pet dogs. Healthy pet dogs were recruited to wear an Actical activity monitor on their collars continuously for 2 weeks in their home environment. At least fifteen dogs were enrolled in each of the following weight ranges: <10, 10–20, 21–30, 31–40, >40 kg and their age, sex and reproductive status recorded. Each dog's intensity of activity for each minute of recording was classified using the total counts for that minute and our pre-established cut-points. The percentage of time dogs spent in sedentary, light or moderate/vigorous activity each day was calculated. Median total daily activity counts and median percentages of time dogs spent in activities of differing intensity were used for the analysis. Associations between signalment characteristics and activity parameters were evaluated with the Mann–Whitney test, the Kruskal–Wallis test and Spearman rank correlations. Ninety-eight dogs were enrolled with ≥17 dogs in each weight category. Time that dogs were sedentary correlated positively with age (*r* 0·50, uncorrected *P* < 0·001), while the median total daily activity count (*r* −0·47, uncorrected *P* < 0·001), time spent in light (*r* −0·46, uncorrected *P* < 0·001) and more vigorous activity (*r* −0·50, uncorrected *P* < 0·001) were negatively associated with age. No other significant associations between signalment and activity parameters were found. The lack of differences in activity across weight categories may reflect the impact of lifestyle negating any potential behavioural differences across breeds.

Canine daily energy requirements have been principally investigated using data collected from dogs kennelled in a controlled environment^(^^1^^)^. Use of kennelled dogs allows for control of variables such as diet, energy intake and body weight. However, the conditions under which kennelled dogs are housed differ in a number of respects from those of pet dogs. For example, the housing of pet dogs can vary substantially among individual households and the kinds of activities in which pet dogs participate are likely to be much more variable and uncontrolled in comparison to dogs kennelled in a controlled environment and are likely influenced by the activities of their owners. There is considerable variation among individuals in the daily maintenance energy requirement of healthy adult dogs. This is due to differences in metabolism, body composition and lifestyle. It may also be influenced by signalment factors including sex, reproductive status, age and breed.

We and others have been investigating an accelerometer-based device that can continuously measure the intensity, frequency and duration of movement for extended periods for use in monitoring activity in pet dogs (Actical, Mini Mitter Inc)^(^^2^^–^^9^^)^. We have previously reported that there is no significant impact of signalment or body conformation on the average activity counts delivered by this monitor when the activity is well controlled and all dogs perform the same movements^(^^2^^)^. Furthermore, we found that a 7-d data collection period provides relatively stable estimates of activity in dogs while including the days with the highest potential for changes in activity to occur (weekends)^(^^3^^)^. We have also shown that an activity monitor can be used to differentiate the intensity of activity of pet dogs through the establishment of activity count cut-points that can be used to discriminate among sedentary, light and more vigorous activities^(^^4^^)^. The ability to quantify activity level in free-living pet dogs and the factors that influence activity could lead to a better understanding of the energy expenditure of pet dogs and the ability to more accurately predict daily energy needs. The aim of the present study was to use accelerometry to investigate associations between signalment and activity of free-living pet dogs.

## Materials and methods

### Dogs

The protocol used in this investigation was in accordance with University of Pennsylvania and United States guidelines for the care and use of animals and was fully approved by the University of Pennsylvania Institutional Animal Care and Use Committee. Written informed consent was obtained from owners of all participating dogs prior to enrolment. Pet dogs belonging to the staff and students of the School of Veterinary Medicine of the University of Pennsylvania were recruited to enrol in this study. For inclusion, the dog had to be in good health based upon medical history and physical examination and in optimal body condition (defined as greater than or equal to a body condition score of 4/9 but less than a body condition score of 6/9) and there could not be any anticipated changes in the household's typical schedule during the data collection period (e.g. vacation, boarding, etc.). If malfunction or removal of the activity monitor during the study period occurred, the dog was removed from study. To ensure inclusion of a diverse cohort, at least fifteen dogs were recruited in each of the following five weight ranges: <10, 10–20, 21–30, 31–40, >40 kg; however, a power calculation was not performed. In each case, signalment data were recorded including age (in years rounded to the nearest half year), sex, reproductive status and breed.

### Experimental design

In all cases, the Actical Activity Monitor, an omnidirectional accelerometer-based device that measures continuously the intensity, frequency and duration of movement, was used. A detailed description of the monitor and how it works is reported elsewhere^(^^5^^)^. The watch-sized monitor was attached to the collar of each dog and positioned ventrally on the neck. The dog's weight was determined using a single calibrated walk-on scale (Arlyn Scales) and recorded. The dog wore the monitor continuously for 2 weeks in its routine home environment at the end of which time it returned to be weighed and for collection of the monitor. The accelerometer data epoch was set at 1 min to permit data collection over an extended period of time.

### Data analysis

Each dog's intensity of activity for each minute of recording was classified using the total counts for that minute and our pre-established cut-points^(^^2^^,^^4^^)^. The percentage of time dogs spent in sedentary, light or moderate/vigorous activity each day was calculated. Median total daily activity counts and median percentages of time dogs spent in activities of differing intensity were used for the analysis. Because activity data were not normally distributed, nonparametric methods of analysis were used. Associations between sex or reproductive status and activity parameters were evaluated with the Mann–Whitney test, between weight category and activity parameters with the Kruskal–Wallis test, and between age, weight and activity parameters with Spearman rank correlation. All tests were two-sided. As twenty comparisons were made between the four activity parameters and five signalment variables, a multiple comparisons correction was applied to the uncorrected overall critical *P* value of 0·05. The corrected overall critical *P* value was 0·003. All analyses were performed using commercially available software (Stata version 8, StataCorp, College Station, Tex, USA).

## Results

One hundred dogs were recruited and ninety-eight dogs, ranging in age from 1 to 14 years (median age = 3 years), were included in the analysis with at least seventeen dogs in each weight category (<10 kg, *n* 21; 10–20 kg, *n* 20; 21–30 kg, *n* 21; 31–40 kg, *n* 18; >40 kg, *n* 17). No dogs dropped out of the study once they were recruited. Two of the recruited dogs were excluded from the analysis, one because the collar with the affixed monitor came off of the dog for an extended period during the 2-week-monitoring period and one because the dog's weight was not recorded at the time of enrolment into the study. There were dogs representing thirty-three different breeds as well as thirty-eight mixed breed dogs included; however, no single breed was represented by more than four individuals. Table 1 shows the data for the associations among activity parameters and sex or reproductive status. Table 2 shows the data for the associations among activity parameters and weight categories. No significant associations were found. The median percentage time that dogs were sedentary was positively correlated with age (*r* 0·50, uncorrected *P* < 0·001), while the median total daily activity count (*r* −0·47, uncorrected *P* < 0·001), median percentage of time spent in light (*r* −0·46, uncorrected *P* < 0·001) and moderate/vigorous activity (*r* −0·50, uncorrected *P* < 0·001) were negatively associated with age. No other significant associations between signalment and activity parameters were found.

**Table 1.** Associations between sex or reproductive status and activity (Median values with ranges)

	Sex					Reproductive status				
	Male (*n* 54)		Female (*n* 44)			Intact (*n* 15)		Neutered (*n* 83)		
	Median	Range	Median	Range	*P*	Median	Range	Median	Range	*P*
Median percentage of time sedentary	87	65–95	87	66–94	0·85	86	65–93	87	66–95	0·55
Median percentage of time light	11	4–31	11	6–25	0·71	13	6–31	11	4–27	0·64
Median percentage of time moderate/vigorous	2	0–13	2	0–8	0·81	2	0–7	2	0–13	0·37
Median total counts	194 892	53 271–2 599 823	206 171	68 643–619 896	0·98	204 494	109 149–545 070	191 320	53 271–2 599 823	0·58

**Table 2.** Associations between weight category and activity (Median values with ranges)

	Weight category										
	<10 kg (*n* 21)		10–20 kg (*n* 20)		21–30 kg (*n* 22)		31–40 kg (*n* 18)		>40 kg (*n* 17)		
	Median	Range	Median	Range	Median	Range	Median	Range	Median	Range	*P*
Median percentage of time sedentary	87	76–92	85	66–94	87	72–93	86	76–92	89	65–95	0·55
Median percentage of time light	11	6–19	13	6–25	10	6–22	12	7–21	10	4–31	0·62
Median percentage of time Moderate/vigorous	2	0–5	3	0–8	2	0–13	1	0–7	1	0–5	0·27
Median total counts	204 494	130 096–336 219	229 731	68 643–619 896	200 044	94 978–2 599 823	203 053	90 728–545 070	133 920	532 071–520 621	0·39

## Discussion

Using acclerometry to monitor activity of pet dogs, we found that the amount of time that dogs were sedentary correlated positively with age while the median total daily activity count and time spent in light and more vigorous activity were negatively associated with age. No other significant associations between signalment and activity parameters were found.

The daily energy requirement of kennelled dogs can be approximated under controlled conditions with reasonable accuracy. The degree that the predictive equations for canine daily energy requirement that are based on data from kennelled dogs reflect the requirements of pet dogs in a home environment is open to question as there has been little published information regarding the pet population^(^^1^^)^. Physical activity, along with the metabolic demands of basic life processes and energy expended as necessary for thermoregulation and assimilation of nutrients from the diet, accounts for the daily maintenance energy requirement of a healthy adult dog and can show considerable variation among individuals. In recent years, accelerometry has shown promise as a means of quantifying physical activity in dogs^(^^2^^–^^9^^)^. In the present investigation, our aim was to look for associations between signalment and activity parameters in free-living pet dogs. If a strong association between activity and one or more aspects of signalment could be found, this information would be helpful for providing more accurate feeding recommendations for pet dogs.

In the present investigation aging was associated with a greater percentage of time sedentary and a smaller percentage of time spent in light or moderate/vigorous activities. These findings are consistent with those reported from an investigation using accelerometry in kennelled beagle dogs. In that study, higher total activity counts were recorded in young (1–4 years) *v.* older (9–14 years) dogs^(^^6^^)^.

We did not find any other statistically significant associations between signalment and activity parameters in this population. Specifically with regard to body weight, there were no significant associations with activity regardless of whether the data were analysed by weight category or for the entire data set of all ninety-eight dogs. This finding is contrary to the perception that certain dog breeds are more or less active. There are limitations to the present study. Although a wide range of body weights and breeds were included in our population, the sample size was not based on a power calculation and no single breed was represented by more than four individuals. It is possible that the lack of differences in activity across weight categories may reflect the impact of lifestyle negating potential behavioural differences across breeds (although, in this investigation we did not attempt to gather and correlate specific information about how the participating dogs were housed or exercised due to the extended interval of time that data were collected over).

In summary using acclerometry in a cohort of pet dogs, of the signalment parameters studied, only age was found to be associated with activity. The present study should be instrumental in insuring design for sufficient power in the future investigations encompassing greater representation of popular breeds. Inclusion of characteristics of the participant's lifestyle to allow investigation of the impact of environment and husbandry is also warranted.
